# Vaccination is associated with reduced mortality rates after surgery for hip fractures in the setting of recent COVID-19 infection: an observational study from the Kaiser Permanente Northern California Database

**DOI:** 10.2340/17453674.2026.45696

**Published:** 2026-05-08

**Authors:** Aidan T Morrell, Ryland P Kagan, Mackenzie Kelly, Graham J Dekeyser, Andrew L Avins, Lusine X Gigoyan, John S Cox

**Affiliations:** 1 Department of Orthopaedics and Rehabilitation, Oregon Health & Science University, Portland, OR; 2 Division of Research, The Permanente Medical Group, Pleasanton, CA; 3 Department of Orthopedic Surgery, The Permanente Medical Group, Walnut Creek, CA, USA

## Abstract

**Background and purpose:**

Limited data exist on mortality and venous thromboembolism (VTE) risk in hip-fracture patients with recent COVID-19 infection. We aimed to examine (i) the association between vaccination status and mortality risk, (ii) whether infection timing is associated with mortality risk, and (iii) whether recent infection is associated with an increase in postoperative VTE risk.

**Methods:**

Adult Kaiser Permanente Northern California members undergoing hip-fracture surgery (2020–2022) were identified. Patients with varying vaccination statuses and SARS-CoV-2 infection histories within 6 months preoperatively were analyzed. Multivariable regression models were adjusted for demographics, comorbidities, and COVID-19 status to calculate risk ratios. The primary outcome was 90-day mortality; the secondary outcome was 90-day VTE.

**Results:**

3,674 patients were included. Unvaccinated or partially vaccinated patients with COVID-19 within 6 months preoperatively had a 4.49-fold higher 90-day mortality risk than fully vaccinated patients (95% confidence interval [CI] 3.72–5.42). Among COVID-positive patients, shortening of the interval from infection to surgery from 6 months to 6 weeks was associated with increased 90-day mortality risk of approximately 53% (CI 1.29–1.97). Additionally, more recent infection (within 6 months) was associated with a 3.14-fold higher postoperative VTE risk (CI 1.10–8.98).

**Conclusion:**

COVID-19 vaccination is associated with a reduction in the mortality risk among hip-fracture patients with recent infection. Shorter infection-to-surgery intervals are associated with increased mortality risk, and recent infection is associated with higher VTE risk.

Coronavirus disease 2019 (COVID-19) is a respiratory disease caused by the SARS-CoV-2 virus. As of January 2025, over 1.2 million people have died of COVID-19 in the United States [1]. Despite the implementation of vaccine and booster programs, COVID-19 remains highly prevalent around the world [2]. Patients with a hip fracture remain a primary public health concern, often resulting in substantial morbidity, loss of independence, and high rates of institutionalization and mortality [3]. The risk of death after a hip fracture in geriatric patients is high, with rates approximately 5–10% at 30 days, 12–18% at 90 days, and up to 20–30% at 1 year [4-9]. Early evidence from the COVID-19 pandemic suggests that older adults with hip fractures who also contract COVID-19 face a dramatically higher risk of adverse outcomes, with 30-day mortality sometimes exceeding 30% [10,11]. Subsequent research indicates that while the increased risk for early mortality persists, it may not be as severe as initially reported [12-14].

Investigating the associations of COVID-19 infection on outcomes after orthopedic surgery, particularly non-elective surgery, is critical for refining perioperative care strategies and improving outcomes in this high-risk group. Furthermore, the role of COVID-19 vaccination in modifying these risks remains underexplored, with little data on whether vaccinated patients have better recovery trajectories or lower mortality following hip fracture surgery.

The study questions were: (i) What is the association between vaccination status and the risk of mortality among patients undergoing operative treatment for hip fracture with recent COVID-19 infection? (ii) Is the timing of a COVID-19 infection relative to surgery associated with mortality risk in hip-fracture patients undergoing operative treatment? (iii) Is recent COVID-19 infection associated with the risk of postoperative venous thromboembolism (VTE) among patients undergoing operative treatment for hip fracture?

## Methods

### Study design and setting

This study employed a retrospective cohort design using data from the electronic medical record of Kaiser Permanente Northern California (KPNC), which is an integrated health plan providing comprehensive health services to more than 4.5 million patients through a network of 21 medical centers. Except for patients in the highest and lowest income brackets, KPNC membership is broadly representative of the Northern California population and is racially and ethnically diverse [15].

The study is reported according to STROBE guidelines.

### Participants/study subjects

Adult KPNC members (aged 18 years and older) who sustained a hip fracture and underwent surgical fixation between January 26, 2020, and December 31, 2022, were included. The study period was chosen to include the first diagnosed case of COVID-19 in the database, until the present day. Data was obtained from the full spectrum of Kaiser Permanente Northern California (KPNC) electronic health record (EHR) and administrative databases, which comprehensively capture all discrete clinical data generated during patient care within the health system. To enhance completeness, internal EHR data was supplemented with external data sources when applicable, including SARS‑CoV‑2 test results performed at non‑Kaiser facilities and reported to local public‑health departments. Mortality ascertainment was further enhanced through linkage with California state mortality files and the US National Death Index. Continuous health‑plan enrollment for at least 12 months prior to fracture was required to ensure complete capture of baseline exposures and clinical characteristics. Patients managed nonoperatively or who were treated with arthroplasty were excluded. Of the included participants, 15 patients with missing race data were grouped under “Other” to preserve sample size, and 1 with missing BMI was excluded from BMI-related analyses. No other variables had missing data.

### Description of investigation, treatment, or surgery

The primary definition of the exposure variable was a known COVID infection within 6 months prior to the surgery or hip fracture. COVID infection was defined by either a positive PCR test result or a COVID diagnosis in the patient’s EHR. Secondary definitions of exposure were COVID infection within 12 weeks and within 6 weeks of the patient’s hip surgery. To assess the accuracy of COVID‑19 infection ascertainment, we conducted a manual chart review of 100 randomly selected patients classified as COVID‑positive based on the study definition. Two study authors verified laboratory results and clinical documentation within the EHR. This validation demonstrated a positive predictive value of 98% (95% exact confidence interval 92.9–99.7%) for the COVID‑19 infection exposure. The index date was defined as the patient’s date of surgery for the hip fracture. The comparison group consisted of KPNC members from the same cohort who did not have a known COVID infection within the defined exposure period prior to surgery.

### Aftercare

Within KPNC, standard practice for patients undergoing surgical repair of a hip fracture is to provide post-event antithrombotic therapy, typically 40 mg subcutaneous enoxaparin daily, or 81 mg aspirin twice daily for 30 days postoperatively.

### Variables, outcome measures, data sources, and bias

The primary study outcome was all-cause mortality occurring within 90 days of the index date. The secondary outcome was the occurrence of a venous thromboembolism (VTE) event (as either an inpatient or outpatient) within the 90-day window following the index date. A VTE event was defined as an EHR-based diagnosis of a deep venous thrombosis (DVT) or pulmonary embolism (PE). Within KPNC, DVT is typically diagnosed by Doppler ultrasound, and PE by chest computed tomography (CT). Outcome ascertainment for venous thromboembolism and mortality was evaluated through manual chart review of 100 randomly selected patients with an outcome event. Two study authors confirmed DVT and PE diagnoses based on imaging reports (Doppler ultrasound for DVT and chest computed tomography for PE), as well as death documentation when applicable. This validation demonstrated a positive predictive value of 97% (95% exact confidence interval 91.4–99.4%) for the composite outcome.

Sociodemographic and clinical variables included age, sex, self-reported race and ethnicity, Neighborhood Deprivation Index (NDI), body-mass index (BMI), surgery class, COVID vaccination status, and prior VTE history at any time. The NDI is a validated composite variable ranging from –5 to +5 with more positive values indicating increasing neighborhood deprivation (e.g., poverty and unemployment) [16]. NDI quartiles were calculated using the full KPNC population. Surgical procedures were categorized as either urgent or elective. For cases initially classified as elective, chart reviews were conducted to verify whether the surgery was performed for the acute treatment of a hip fracture. Only cases confirmed as urgent—either by initial classification or through chart review—were included in the analysis. Elective procedures not related to acute hip fracture management were excluded. BMI was determined from the closest measurement within 365 days of surgery and categorized according to the Centers for Disease Control and Prevention’s established cutoffs: <18 (underweight), 18–24.9 (normal weight), 25–29.9 (overweight), and ≥30 (obese) [17].

COVID vaccination status was defined as follows: individuals were considered fully vaccinated if they had received both doses of either Pfizer or Moderna vaccines, or 1 dose of the Janssen COVID vaccine, within 12 months prior to the date of surgery. Those who had received only 1 dose of either the Pfizer or Moderna vaccine within the same 12-month period were categorized as partially vaccinated. Individuals who did not meet either of these criteria were classified as unvaccinated. The numbers of partially vaccinated patients were so small that these individuals were grouped with the unvaccinated group. Prior history of VTE was defined as the presence of a VTE diagnosis in the EHR at any time prior to the surgery.

### Statistics

All analyses were focused on identifying independent associations among the predictor and outcome variables without seeking to determine causality or making out-of-sample predictions. Descriptive statistics were used to summarize patient characteristics. Continuous variables were reported as means with standard deviations (SDs) or medians with interquartile ranges (IQRs), and categorical variables as frequencies and percentages. Fisher’s exact tests were used for univariable comparisons when sample sizes were small.

For adjusted analyses, we employed modified Poisson regression using generalized estimating equations (GEE) with a Poisson distribution and robust (sandwich) standard errors to estimate risk ratios (RRs) and their associated 95% confidence intervals (CI) for binary outcomes. This approach was selected for its ability to directly estimate relative risks in the context of non-rare outcomes and is supported by prior methodological work by Barros and Hirakata [18] and Zou [19]. All variables included in each multivariable model are listed and accounted for in each table.

Unadjusted analyses were conducted using Fisher’s exact tests or univariable Poisson regression as appropriate. All statistical analyses were performed using R version 4.2.1 (R Foundation for Statistical Computing, Vienna, Austria), with 2-sided tests and an alpha level of 0.05 to define statistical significance.

As all reported measures of association were derived from an observational, retrospective cohort design using existing clinical data, all such estimates should be viewed as associative only; no causality should be assumed.

### Ethics, funding, and potential conflicts of interest

This study was conducted under approval by the Kaiser Permanente Northern California Institutional Review Board (Approval #2056765). Due to regulatory restrictions, data sharing is not possible; however, requests for sub-analyses may be considered. The study was internally funded by the Permanente Medical Group’s Delivery Science and Applied Research Program. The funding source had no role in data interpretation, result reporting, manuscript preparation, or the decision to publish. All authors certify that they have no commercial associations that could present a conflict of interest related to this work. Complete disclosure of interest forms, in accordance with ICMJE guidelines, are available on the article page, doi: 10.2340/17453674.2026.45696

## Results

### Study population

**Figure 1:**
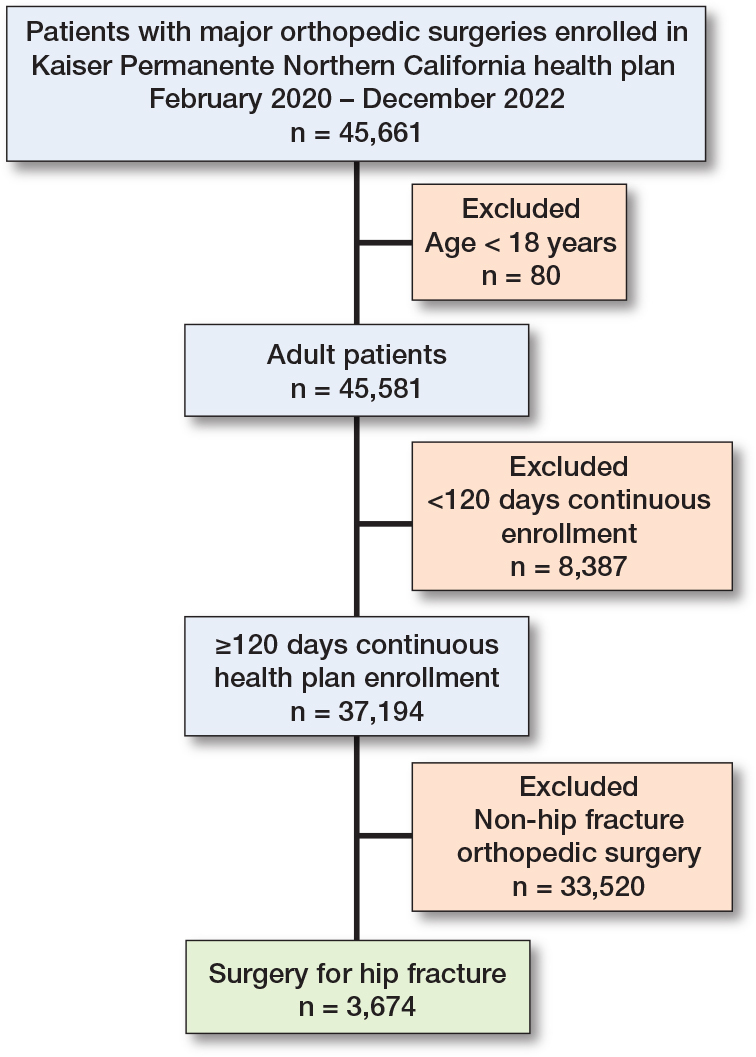
Patient study flow diagram.

3,674 eligible patients who underwent surgical repair of a hip fracture were identified from the Kaiser Permanente Northern California database between January 2020 and December 2022 (Figure 1). The cohort was predominantly older, with 3,233 patients (88%) aged 65 years or older, and demonstrated a female predominance (2,321 women [63%], 1,353 men [37%]). The mean age was 78 years (SD 9), and the age distribution was as follows: 5% aged 18–54 years, 38% aged 55–74 years, and 57% aged 75 years or older. The majority of patients were White, with additional representation from Black, Asian, Hispanic, and other racial or ethnic groups (Table 1). Most patients (84%) were fully vaccinated against COVID-19 at the time of their hip fracture, while 16% were either unvaccinated or partially vaccinated. Surgical procedures were classified as urgent in nearly all cases, consistent with the nonelective nature of hip-fracture repair. The median time from emergency department (ED) arrival to time of surgery was 19.2 hours overall (IQR 11.5–14.5; range 1.2–46.0); there was no significant difference in time to surgery between vaccinated and unvaccinated patients (18.4 vs 18.6 hours, respectively, P = 0.7).

**Table 1:** Sociodemographics, clinical characteristics, and outcomes of patients with surgical treatment of hip fracture by preoperative COVID vaccination status. Values are count (%)

Characteristic	Total	Fully vaccinated	Unvaccinated
	N = 3,674	n = 3,094	n = 580 ^a^
Age at surgery			
< 55	160 (4.4)	137 (4.4)	23 (4.0)
56–65	271 (7.4)	227 (7.3)	44 (7.6)
≥ 65	3,243 (88)	2,730 (88)	513 (88)
Biological sex			
Female	2,557 (70)	2,167 (70)	390 (67)
Male	1,117 (30)	927 (30)	190 (33)
Race/ethnicity			
White	2,835 (77)	2,395 (77)	440 (76)
Hispanic	383 (10)	311 (10)	72 (12)
Black	129 (3.5)	96 (3.1)	33 (5.7)
Asian	283 (7.7)	253 (8.2)	30 (5.2)
Other/unknown	44 (1.2)	39 (1.3)	5 (0.9)
NDI quartiles			
Q1 (low deprivation)	976 (27)	855 (28)	121 (21)
Q2	1,198 (33)	1,009 (33)	189 (33)
Q3	894 (24)	748 (24)	146 (25)
Q4 (high deprivation)	606 (16)	482 (16)	124 (21)
Baseline BMI			
< 18.5	377 (10)	291 (9.4)	86 (15)
18.5–25	1,805 (49)	1,495 (48)	310 (53)
26–30	993 (27)	865 (28)	128 (22)
≥ 30	498 (14)	442 (14)	56 (9.7)
Unknown	(< 1)	(< 1)	(0)
Urgent surgery	3,674 (100)	3,094 (100)	580 (100)
COVID preoperatively			
within 6 months	193 (5.3)	165 (5.3)	28 (4.8)
within 12 weeks	102 (2.8)	85 (2.7)	17 (2.9)
within 6 weeks	73 (2.0)	62 (2.0)	11 (1.9)
Within 90 days of surgery			
VTE	27 (0.7)	20 (0.6)	7 (1.2)
All-cause mortality	390 (11)	202 (6.5)	188 (32)

a The numbers of partially vaccinated patients were so small that these individuals were grouped with the unvaccinated group.

BMI = body mass index; NDI = Neighborhood Deprivation Index; VTE = venous thromboembolism.

Patients were represented across all quartiles of the Neighborhood Deprivation Index (NDI), a measure of socioeconomic disadvantage. Unvaccinated individuals were more likely to live in the most deprived areas (Q4: 21.4%) and less likely to live in the affluent areas (Q1: 20.9%) compared with fully vaccinated individuals (Q4: 15.6%, Q1: 27.6%). This difference was statistically significant (chi-square [χ²] = 18.60, P < 0.001).

### Association between vaccination status and risk of mortality among patients undergoing operative treatment for hip fracture with recent COVID-19 infection

390 individuals (10.6%) died within 90 days of surgery. Mortality was significantly higher among unvaccinated or partially vaccinated patients (188/580, 32.4%) compared with fully vaccinated patients (202/3,094, 6.5%) (see Table 1). Among patients with a COVID-19 infection within 6 months prior to surgery, those who were unvaccinated or partially vaccinated had a 4.49-fold higher risk of 90-day mortality compared with fully vaccinated patients (CI 3.72–5.42). COVID vaccination status showed the strongest association with mortality across all time points assessed (Tables 2 and 3).

**Table 2:** Risk of all-cause mortality within 90 days of surgery for patients undergoing surgical repair of hip fracture. Exposure is any COVID infection within 6 months, 12 weeks, and 6 weeks of surgery (univariable analysis)

COVID infection	Total	Died (%)	Risk ratio (CI)	P value
Within 6 months				
Yes	193	26 (13)	_1.29 (0.89–1.87)_	0.2
No	3,481	364 (10)		
Within 12 weeks				
Yes	102	18 (18)	_1.69 (1.10–2.61)_	0.03
No	3,572	372 (10)		
Within 6 weeks				
Yes	73	15 (21)	_1.97 (1.24–3.13)_	0.01
No	3,601	375 (10)		

CI = 95% confidence interval.

**Table 3:** Risk of all-cause mortality within 90 days of surgery for patients undergoing surgical repair of hip fracture. Exposure is any COVID infection within 6 months, 12 weeks, and 6 weeks of surgery (multivariable analysis by Poisson regression)

Characteristic	within 6 months		COVID infection within 12 weeks		within 6 weeks	
	Risk ratio (CI)	P value	Risk ratio (CI)	P value	Risk ratio (CI)	P value
COVID infection						
No COVID	Reference					
COVID	1.22 (0.80–1.84)	0.4	1.59 (1.04–2.41)	0.03	1.83 (1.15–2.89)	0.01
COVID vaccination						
Fully vaccinated	Reference					
Unvaccinated	4.49 (3.72–5.42)	< 0.001	4.36 (3.63–5.24)	< 0.001	4.37 (3.64–5.25)	< 0.001
Age						
< 55	Reference					
56–65	0.67 (0.23–1.97)	0.5	0.77 (0.28–2.09)	0.6	0.78 (0.29–2.12)	0.6
≥ 65	2.50 (1.16–5.40)	0.02	2.46 (1.14–5.32)	0.02	2.47 (1.15–5.35)	0.02
Sex						
Female	Reference					
Male	1.38 (1.14–1.66)	< 0.001	1.36 (1.13–1.63)	< 0.001	1.36 (1.13–1.63)	< 0.001
Race/ethnicity						
White	Reference					
Hispanic	1.02 (0.76–1.36)	> 0.9	1.02 (0.77–1.34)	0.9	1.01 (0.77–1.33)	> 0.9
Black	0.65 (0.41–1.02)	0.06	0.63 (0.40–1.00)	0.049	0.63 (0.40–0.99)	0.04
Asian	0.96 (0.67–1.39)	0.8	0.87 (0.59–1.27)	0.5	0.86 (0.59–1.26)	0.4
Other/unknown	1.48 (0.65–3.38)	0.3	1.33 (0.57–3.10)	0.5	1.35 (0.58–3.13)	0.5
NDI quartiles						
Q1 (low deprivation)	Reference					
Q2	0.98 (0.78–1.20)	0.9	0.96 (0.76–1.20)	0.7	0.96 (0.76–1.20)	0.7
Q3	0.92 (0.72–1.19)	0.5	0.92 (0.72–1.17)	0.5	0.92 (0.72–1.17)	0.5
Q4 (high deprivation)	0.88 (0.65–1.19)	0.4	0.87 (0.65–1.17)	0.4	0.88 (0.66–1.17)	0.4
Baseline BMI						
< 18.5	Reference					
18.5–25	0.77 (0.59–1.00)	0.049	0.76 (0.59–0.97)	0.03	0.75 (0.59–0.97)	0.03
26–30	0.65 (0.48–0.87)	0.004	0.66 (0.49–0.88)	0.005	0.66 (0.49–0.88)	0.005
≥ 30	0.47 (0.31–0.72)	< 0.001	0.50 (0.34–0.75)	< 0.001	0.50 (0.34–0.75)	< 0.001
Surgery class						
Elective	Reference					
Urgent	1.08 (0.61–1.92)	0.8	1.17 (0.65–2.09)	0.6	1.16 (0.65–2.09)	0.6

For abbreviations, see Table 1 and CI = 95% confidence interval.

#### Timing of a COVID-19 infection relative to surgery associated with mortality risk in hip-fracture patients undergoing operative treatment

Mortality risk increased as the time between COVID-19 infection and surgery decreased. The adjusted risk ratio rose from 1.22 (CI 0.80–1.84) for infections within 6 months to 1.83 (CI 1.15–2.89) for infections within 6 weeks (Tables 2 and 3). Statistically significant associations were observed for the 12-week and 6-week exposure windows in both univariable and multivariable models.

#### Recent COVID-19 infection associated with the risk of postoperative VTE among patients undergoing operative treatment for hip fracture

27 patients (0.7%) experienced a postoperative VTE within 90 days (Table 4). Patients with a COVID-19 infection within 6 months had a higher risk of VTE (RR 3.14, CI 1.10–8.98) compared with those without recent infection (Table 4). This association persisted for 12-week and 6-week exposure periods but did not reach statistical significance at 6 weeks (P = 0.1; Table 4). Multivariable analysis was not performed due to limited numbers.

**Table 4:** Risk of venous thromboembolic (VTE) event within 90 days of surgery for patients undergoing surgical treatment of hip fracture. a Exposure is any COVID infection within 6 months, 12 weeks and 6 weeks of surgery (univariable analysis)

COVID infection	Total	VTE (%)	Risk ratio (CI)	P value
Within 6 months of surgery				
COVID infection	193	4 (2.1)	_3.14 (1.10–8.98)_	0.05
No COVID infection	3,481	23 (0.7)		
Within 12 weeks of surgery				
COVID infection	102	4 (3.9)	_6.09 (2.15–17.3)_	0.006
No COVID infection	3,572	23 (0.6)		
Within 6 weeks of surgery				
COVID infection	73	2 (2.7)	_3.95 (0.95–16.4)_	0.1
No COVID infection	3,601	25 (0.7)		
Total	3,674	27 (0.7)		

For abbreviations, see Table 1 and CI = 95% confidence interval.

## Discussion

We examined the association of vaccination status with the risk of mortality and VTE in the setting of recent COVID-19 infection. We demonstrated that recent COVID-19 infections are associated with an increased risk of mortality and VTE, raising the possibility that vaccination may be a modifiable protective risk factor. Additionally, in our cohort, unvaccinated patients were more likely to reside in socioeconomically disadvantaged neighborhoods, as reflected by higher representation in the most deprived NDI quartile. This finding aligns with broader socioeconomic patterns in healthcare access and fracture risk. Individuals in more deprived neighborhoods may experience limited healthcare access, lower health literacy, and vaccine hesitancy. These structural factors could partially explain lower vaccination uptake and worse outcomes observed in this population.

Our findings are consistent with and extend our prior analysis of elective total hip and knee arthroplasty performed within the same health system and study period [20]. In that arthroplasty cohort, recent COVID‑19 infection was associated with increased 90‑day mortality when infection occurred within 6–12 weeks preoperatively, while no significant increase in postoperative VTE risk was observed; notably, no 90‑day deaths occurred among fully vaccinated patients with recent infection. In the present hip fracture cohort, we similarly observed higher mortality with more recent infection and a strong protective association with vaccination. However, unlike elective arthroplasty, recent infection in hip fracture patients was also associated with an increased risk of postoperative VTE. These differences likely reflect the nonelective nature of hip fracture surgery, greater patient frailty, and reduced opportunity for physiologic recovery and perioperative optimization compared with elective arthroplasty.

### Limitations

First, we did not include COVID-positive patients with hip fractures who were managed nonoperatively, accounting for 9.3%. Although including these patients would likely affect this study’s mortality rate, our aim was to evaluate outcomes for patients who underwent operative treatment for their hip fractures. Second, we did not stratify patients by severity of COVID-19 infection. Given that severity of COVID-19 symptoms is impacted by vaccination status [21], sensitivity analyses in future studies that can capture both variables would be warranted to clarify their relationship. Third, our dataset included all patients diagnosed with hip fracture in the ED, even if they were later transferred to non-Kaiser facilities for surgery or had fracture types typically managed nonoperatively (e.g., isolated greater trochanter or nondisplaced periprosthetic fractures). This may overestimate the proportion of nonoperative cases, but a detailed chart review to fully distinguish these scenarios was beyond the scope of this study. Fourth, patients with hip fractures treated with arthroplasty were excluded from the analysis to focus on internal fixation procedures. This exclusion may limit the generalizability of our findings to all surgically treated hip fractures. Finally, our findings are subject to limitations inherent in large databases including selection bias, and our ability to characterize this population at a more granular level to account for additional covariates such as postoperative VTE prophylaxis is limited, albeit similar to other large observational studies.

### Vaccination status and the risk of mortality among patients undergoing operative treatment for hip fracture with recent COVID-19 infection

Our study demonstrates that vaccination against COVID-19 is associated with reduced mortality among patients with recent COVID-19 infections when compared with those who were incompletely or not vaccinated. Furthermore, COVID-19 vaccination status was more strongly associated with mortality than all other variables included in our study. These data are consistent with a study in the United Kingdom that also found lower mortality among vaccinated COVID-positive patients undergoing surgery for hip fracture [22]. In addition, a study based in New York from a multi-hospital healthcare system reported a higher 30-day mortality rate among their pre-vaccine COVID-positive patients compared with the post-vaccine cohort; however, this association did not reach statistical significance. Of note, only 35% of the post-vaccine cohort had been completely vaccinated, which may have diluted the treatment effect [23].

### Timing of COVID-19 infection relative to surgery associated with mortality risk in hip-fracture patients undergoing operative treatment

We found that the risk of mortality increased as the time between COVID-19 infection and surgery decreased. Increased perioperative risks for patients who are COVID-positive have already been well established [24,25], though there is no consensus on the ideal time for surgery [26,27]. For urgent or non-elective procedures, it is important to understand the impact of COVID-19 infection on a patient’s risk profile primarily to help guide consent and decision-making steps with a patient and their family. Additionally, more recent studies [28,29] suggest that though this patient cohort may be at an elevated risk of short-term mortality and other adverse outcomes, the overall prognosis may be better than what was suggested by the first collaborative studies from early in the pandemic [12-14].

While our study focuses on COVID-19, the findings may have broader implications for understanding how infectious diseases impact surgical outcomes in elderly patients. Prior research has shown that concurrent infections at the time of hip fracture surgery are significantly associated with postoperative mortality. For instance, a study by Sheikh et al. [30] found that a concomitant chest infection was associated with a 3.7-fold increase in 30-day mortality. Similarly, preoperative pneumonia has been linked to a 30-day mortality rate of 11.9% compared with 5% in uninfected patients, and a 1-year mortality rate of 33.9% vs 16.3% [31]. These findings align with our observation that recent COVID-19 infection—particularly when close to the time of surgery—is associated with a substantially increased mortality risk.

However, not all infections confer the same risk. For example, a study by Crouser et al. [32] found no significant difference in 30-day mortality among patients with urinary tract infections at the time of surgery. A systematic review by Guerado et al. [33] further supports the notion that the type and severity of infection, as well as the timing relative to surgery, are critical factors in determining outcomes. As new infectious threats emerge, our findings underscore the importance of infection timing and vaccination status in mitigating surgical risk in this vulnerable population.

### Recent COVID-19 infection associated with the risk of postoperative VTE among patients undergoing operative treatment for hip fracture

A recent COVID-19 infection was associated with a higher risk of VTE among patients undergoing surgical management for a hip fracture. This is consistent with a systematic review of retrospective cohort studies, which reported a pooled odds ratio of 2.8 (CI, 1.1–7.1, P = 0.03) comparing similar patients [14]. Given the low incidence of this complication among this population, further studies or sub-analyses from large registry databases may clarify this risk in the future. It would be especially important to account for anticoagulant treatment protocols.

### Conclusions

We show that a recent COVID-19 infection among patients undergoing surgery for a hip fracture is associated with an increased risk of mortality and VTE. Furthermore, COVID-19 vaccination status is strongly associated with a lower mortality risk. Future research should explore whether implementing COVID-19 vaccination programs targeted at older adults can reduce adverse outcomes, including VTE and mortality, in this high-risk group.

### Supplementary data

Tables 1–3 (sensitivity analyses) are available as supplementary data on the article page, doi: 10.2340/17453674.2026.45696

## Supplementary Material



## Notes

ATM: conceptualization, analyzing and interpreting data, drafting and revising the manuscript. GD, RK: conceptualization, analyzing and interpreting data, revising the manuscript. MK: interpreting data, revising the manuscript. AA, LG: analyzing and interpreting data, revising the manuscript. JC: conceptualization, analyzing and interpreting data, drafting and revising the manuscript; guarantor of the work.

Handling co-editors: Cecilia Rogmark and Philippe Wagner

*Acta* thanks Sunniva Leer-Salvesen for help with peer review of this manuscript.
