# Supplementary material for: Vaccination is associated with reduced mortality rates after surgery for hip fractures in the setting of recent COVID-19 infection: an observational study from the Kaiser Permanente Northern California Database
Source: Acta Orthop. 2026 May 8;97:286–93. doi: 10.2340/17453674.2026.45696 (PMC13154000; doi:10.2340/17453674.2026.45696)
Supplement: Supplementary file 1 [file ActaO-97-45696-s1.pdf]

<u>Tables below show a sensitivity analysis for the age ≥60 years-old subgroup.</u>

Of our study cohort (N = 3,674), 93.2% (n = 3,426) were aged at least 60 years.

Table 2A-1. Exposure is any COVID infection within 6 months of surgery (univariable analysis) (sensitivity analysis for the age ≥60 years-old subgroup)

	**Died, n (%)**	**Alive, n (%)**	**Total**
**Risk ratio 1.29, CI 0.88–1.87, P = 0.2**			
Covid infection	25 (14.2)	151 (85.8)	176
No Covid infection	359 (11.0)	2,891 (89.0)	3,250
Total	384 (11.2)	3,042 (88.8)	3,426

Table 2A-2. Exposure is any COVID infection within 6 months of surgery (multivariable analysis) (sensitivity analysis for the age ≥60 years-old subgroup)

**Characteristic**	**Risk ratio** [a]	**CI**	**P value**
**Covid infection**			
No Covid 6 months	—	—	
Covid 6 months	1.26	0.87–1.83	0.2
**Covid vaccination**			
Fully vaccinated	—	—	
Unvaccinated	4.36	3.62–5.24	<0.001
**Sex**			
Female	—	—	
Male	1.37	1.14–1.65	<0.001
**Race / ethnicity**			
White	—	—	
Hispanic	0.98	0.74–1.30	0.9
Black	0.64	0.40–1.01	0.056
Asian	0.85	0.58–1.26	0.4
Other/unknown	1.26	0.54–2.96	0.6
**NDI quartiles**			
Q1 (low deprivation)	—	—	
Q2	0.95	0.75–1.19	0.6
Q3	0.91	0.71–1.17	0.5
Q4 (high deprivation)	0.90	0.67–1.19	0.5
**Baseline BMI**			
<18.5	—	—	
18.5–25	0.78	0.60–1.00	0.055
26–30	0.64	0.48–0.86	0.003
≥30	0.51	0.35–0.76	<0.001
**Surgery class**			
Elective	—	—	
Urgent	1.14	0.65–2.01	0.6

[a] Adjusted for the covariates listed in the table.
NDI = Neighborhood Deprivation Index

Table 2B-1: Exposure is any COVID infection within 12 weeks of surgery (univariable analysis) (sensitivity analysis for the age ≥60 years-old subgroup)

	Died, n (%)	Alive, n (%)	Total
**Risk ratio 1.76, CI 1.15–2.70, P = 0.02**			
Covid infection	18 (19.4)	75 (80.6)	93
No Covid infection	366 (11.0)	2,967 (89.0)	3,333
Total	384 (11.2)	3,042 (88.8)	3,426

Table 2B-2. Exposure is any COVID infection within 12 weeks of surgery (multivariable analysis) (sensitivity analysis for the age ≥60 years-old subgroup)

Characteristic	Risk ratio [a]	CI	P value
**Covid infection**			
No Covid 12 weeks	—	—	
Covid 12 weeks	1.60	1.05–2.45	0.03
**Covid vaccination**			
Fully vaccinated	—	—	
Unvaccinated	4.34	3.61–5.22	<0.001
**Sex**			
Female	—	—	
Male	1.37	1.14–1.65	<0.001
**Race / ethnicity**			
White	—	—	
Hispanic	0.99	0.75–1.30	>0.9
Black	0.64	0.40–1.00	0.052
Asian	0.85	0.58–1.25	0.4
Other/unknown	1.26	0.54–2.96	0.6
**NDI quartiles**			
Q1 (low deprivation)	—	—	
Q2	0.94	0.75–1.19	0.6
Q3	0.91	0.71–1.16	0.4
Q4 (high deprivation)	0.89	0.67–1.19	0.4
**Baseline BMI**			
<18.5	—	—	
18.5–25	0.78	0.60–1.00	0.050
26–30	0.64	0.48–0.86	0.003
≥30	0.51	0.34–0.75	<0.001
**Surgery class**			
Elective	—	—	
Urgent	1.15	0.65–2.04	0.6

[a] Adjusted for the covariates listed in the table.

Table 2C-1. Exposure is any COVID infection within 6 weeks of surgery (univariable analysis) (sensitivity analysis for the age ≥60 years-old subgroup)

	Died, n (%)	Alive, n (%)	Total
**Risk ratio 1.97, CI 1.24–3.13, P = 0.01**			
Covid infection	15 (23.1)	50 (76.9)	65
No Covid infection	369 (11.0)	2,992 (89.0)	3,361
Total	384 (11.2)	3,042 (88.8)	3,426

Table 2C-2: Exposure is any COVID infection within 6 weeks of surgery (multivariable analysis) (sensitivity analysis for the age ≥60 years-old subgroup)

Characteristic	Risk ratio [a]	CI	p-value
**Covid infection**			
No Covid 6 weeks	—	—	
Covid 6 weeks	1.92	1.21–3.04	0.006
**Covid vaccination**			
Fully vaccinated	—	—	
Unvaccinated	4.35	3.62–5.23	<0.001
**Sex**			
Female	—	—	
Male	1.37	1.14–1.65	<0.001
**Race / ethnicity**			
White	—	—	
Hispanic	0.98	0.74–1.29	0.9
Black	0.63	0.40–0.99	0.046
Asian	0.85	0.58–1.24	0.4
Other/unknown	1.27	0.54–2.99	0.6
**NDI quartiles**			
Q1 (low deprivation)	—	—	
Q2	0.95	0.75–1.19	0.6
Q3	0.91	0.71–1.17	0.5
Q4 (high deprivation)	0.90	0.67–1.20	0.5
**Baseline BMI**			
<18.5	—	—	
18.5–25	0.77	0.60–1.00	0.048
26–30	0.64	0.48–0.86	0.003
≥30	0.51	0.34–0.75	<0.001
**Surgery class**			
Elective	—	—	
Urgent	1.15	0.65–2.04	0.6

[a] Adjusted for the covariates listed in the table.

Table 3A. Exposure is any COVID infection within 6 months of surgery (univariable analysis) (sensitivity analysis for the age ≥60 years-old subgroup)

	Developed VTE, n (%)	Did not develop VTE, n (%)	Total
**Risk ratio 3.36, CI 1.17–9.64, P = 0.054)**			
Covid infection	4 (2.1)	172 (97.9)	176
No Covid infection	22 (0.7)	3,228 (99.3)	3,250
Total	26 (0.7)	3,400 (99.3)	3,426

Table 3B. Exposure is any COVID infection within 12 weeks of surgery (univariable analysis) (sensitivity analysis for the age ≥60 years-old subgroup)

	Developed VTE, n (%)	Did not develop VTE, n (%)	Total
**Risk ratio 6.52, CI 2.29–18.5, P = 0.005**			
Covid infection	4 (4.3)	89 (95.7)	93
No Covid infection	22 (0.7)	3,311 (99.3)	3,333
Total	26 (0.8)	3,400 (99.2)	3,426

Table 3C. Exposure is any COVID infection within 6 weeks of surgery (univariable analysis) (sensitivity analysis for the age ≥60 years-old subgroup)

	Developed VTE, n (%)	Did not develop VTE, n (%)	Total
**Risk ratio 4.31, CI 1.04–17.9, P = 0.09**			
Covid infection	2 (3.0)	63 (97.0)	65
No Covid infection	24 (0.7)	3,337 (99.3)	3,361
Total	26 (0.8)	3,400 (99.2)	3,426
